# Supplementary material for: Identification of photoperiod-induced specific miRNAs in the adrenal glands of Sunite sheep (Ovis aries)
Source: Front Vet Sci. 2022 Jul 22;9:888207. doi: 10.3389/fvets.2022.888207 (PMC9354845; doi:10.3389/fvets.2022.888207)
Supplement: Supplementary file 1 [file Data_Sheet_1.ZIP › Supplementary file/miRDeep2 file.zip!/miRDeep2 file/Novel_Structure/SP42A2/Novel_96.pdf]

Provisional ID	:	NC_019484.2_42717
Score total	:	2.2
Score for star read(s)	:	-1.3
Score for read counts	:	0
Score for mfe	:	1.9
Score for randfold	:	1.6
Score for cons. seed	:	
Total read count	:	27
Mature read count	:	25
Loop read count	:	0
Star read count	:	2

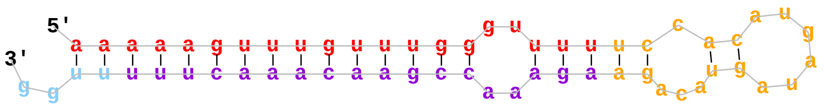

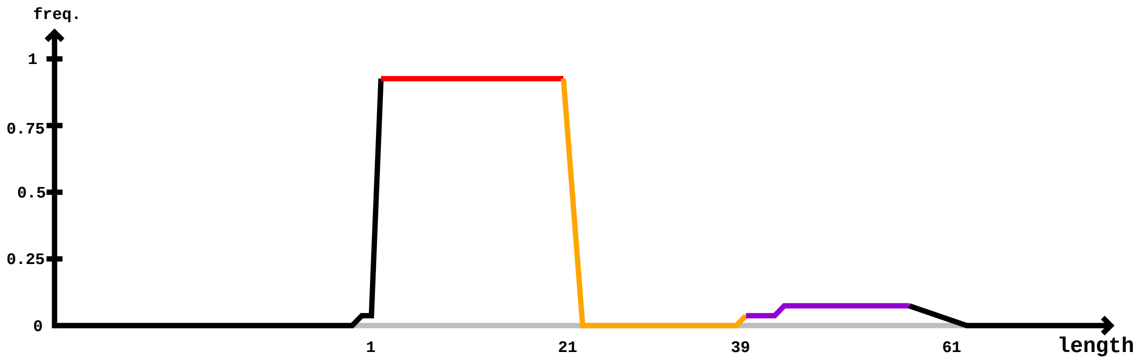

Mature **Star**

```
5'- gggugaggaacucgguacuauugggguuggcaaaaaaguuuguuuggguuuuuccacaugauaguacagaagaaaccgaacaaacuuuuuggcuaauccaauagcagc   -3'    obs
    gggugaggaacucgguacuauugggguuggcaaaaaaguuuguuuggguuuuuccacaugauaguacagaagaaaccgaacaaacuuuuuggcuaauccaauagcagc          exp
    ((((.....))))....((((((((.(((((.((((((((((((((..(((((.((......))...)))))..)))))))))))))).)))))))))))))....   reads    mm    sample
    ...............................caaaaaguuuguuuggguuuu........................................................   1        0     seq
    ................................aaaaaguuCguuuggguuuu........................................................   23       1     seq
    ................................aaaaaguuuguuuggguuuuu.......................................................   1        0     seq
    ......................................................................agaaaccgaacaaacuuu....................   1        0     seq
    ..........................................................................Uccgaacaaacuuuuuggc...............   1        1     seq
```
